# GC/MS Profiling of the Essential Oil and Lipophilic Extract of *Moricandia sinaica* Boiss. and Evaluation of Their Cytotoxic and Antioxidant Activities

**DOI:** 10.3390/molecules28052193

**Published:** 2023-02-27

**Authors:** Shaza H. Aly, Nariman H. Kandil, Roqaya M. Hemdan, Sara S. Kotb, Sara S. Zaki, Omnia M. Abdelaziz, Mohamed M. M. AbdelRazek, Hadia Almahli, Mahmoud A. El Hassab, Sara T. Al-Rashood, Faizah A. Binjubair, Wagdy M. Eldehna

**Affiliations:** 1 Department of Pharmacognosy, Faculty of Pharmacy, Badr University in Cairo (BUC), Cairo 11829, Egypt; 2 Department of Chemistry, University of Cambridge, Cambridge CB2 1EW, UK; 3 Department of Medicinal Chemistry, Faculty of Pharmacy, King Salman International University (KSIU), South Sinai 46612, Egypt; 4 Department of Pharmaceutical Chemistry, College of Pharmacy, King Saud University, P.O. Box 2457, Riyadh 11451, Saudi Arabia; 5 Department of Pharmaceutical Chemistry, Faculty of Pharmacy, Kafrelsheikh University, Kafrelsheikh 33516, Egypt; 6 School of Biotechnology, Badr University in Cairo, Badr City 11829, Egypt

**Keywords:** antioxidant, cytotoxicity, essential oil, GC/MS, *Moricandia sinaica*, molecular docking

## Abstract

The genus *Moricandia* (Brassicaceae) comprises about eight species that were used in traditional medicine. *Moricandia sinaica* is used to alleviate certain disorders such as syphilis and exhibits analgesic, anti-inflammatory, antipyretic, antioxidant, and antigenotoxic properties. Throughout this study, we aimed to figure out the chemical composition of lipophilic extract and essential oil obtained from *M. sinaica* aerial parts using GC/MS analysis, as well as their cytotoxic and antioxidant activities correlated with the major detected compounds’ molecular docking. The results revealed that both the lipophilic extract and the oil were found to be rich in aliphatic hydrocarbons, accounting for 72.00% and 79.85%, respectively. Furthermore, the lipophilic extract’s major constituents are octacosanol, *γ*-sitosterol, *α*-amyrin, *β*-amyrin acetate, and *α*-tocopherol. Contrarily, monoterpenes and sesquiterpenes accounted for the majority of the essential oil. The essential oil and the lipophilic extract of *M. sinaica* showed cytotoxic properties towards human liver cancer cells (HepG2) with IC_50_ values of 126.65 and 220.21 µg/mL, respectively. The lipophilic extract revealed antioxidant activity in the DPPH assay with an IC_50_ value of 2679 ± 128.13 µg/mL and in the FRAP assay, moderate antioxidant potential was expressed as 44.30 ± 3.73 µM Trolox equivalent/mg sample. The molecular docking studies revealed that *ꞵ*-amyrin acetate, *α* -tocopherol, *γ*-sitosterol, and *n*-pentacosaneachieved the best docking scores for NADPH oxidase, phosphoinositide-3 kinase, and protein kinase B. Consequently, *M. sinaica* essential oil and lipophilic extract can be employed as a viable management strategy for oxidative stress conditions and the formulation of improved cytotoxic treatment regimens.

## 1. Introduction

For millennia, the spotlight has been directed toward medicinal plants as a plentiful supply of bioactive compounds, and many of the therapeutic medications currently in use are natural products or compounds derived from plants [1,2,3,4,5]. According to the World Health Organization (WHO), about 80% of the world’s population lives in developing and underdeveloped countries and relies on medicines of natural origin as a remedy for medical ailments [6]. Plants are considered a potential source for the finding of new candidate compounds. Natural plants and their isolated chemicals provide us with a great source of biologically active leads for the development of drugs with benefits both in terms of cost and fewer side effects [1,7,8,9,10,11].

The mustard family, Brassicaceae, comprises approximately 338 genera and 3709 species, which are cultivated presently worldwide with high economic importance since they are widely consumed in human diets all over the world. Moreover, they are used to produce food and oilseed crops, while a number of other varieties are used as ornamental plants (with violet, purple, and white flowers) and noxious weeds [12,13]. The genus *Moricandia* (family: Brassicaceae) has eight species, namely *arvensis*, *foetida*, *foleyii*, *oricandioides*, *nitens*, *sinaica*, *spinosa*, and *suffruticosa,* some of which are used in traditional medicine [14]. These species are distributed all over North Africa, the Mediterranean basin, West Asia, and Southeast Asia [15]. *Moricandia* plants have revealed some significant health properties. For instance, *M. arvensis* leaves, which are frequently used in Tunisian traditional dishes, exhibit beneficial effects in the management of syphilis [16,17]. Additionally, some *M. arvensis* extracts have been shown to have antioxidant and antigenotoxic properties, and they effectively slow the spread of human cancer cells [18,19]. 

One of these species, *Moricandia sinaica*, is native to Saudi Arabia and mainly seen in desert areas such as Western Asia, the Middle East, and Egypt [13,20]. *Moricandia sinaica* is a suffrutescent to suffruticose annual to perennial plant; the leaves are thick, pointed, and vary in shape from oval to oblong-ovate, with pink or white corolla flowers [13]. It is indigenous to the Mediterranean area, Europe, and America, and has therapeutic uses in traditional medicine [20]. To date, there are few investigations that focus on the therapeutic potential or phytoconstituents of *M*. *sinaica*. Crude extracts from the roots, stem, leaves, and shoots of *M. sinaica* were investigated and evaluated for potential antiangiogenic effects in zebrafish embryos [13]. Besides, in vivo, various fractions of *M. sinaica* aerial parts exhibit analgesic, anti-inflammatory, and antipyretic properties [21].

Herein, the current research is primarily focused on the phytochemical investigations of the essential oil and lipophilic extract of *M.sinaica* through GC/MS analysis, besides the cytotoxic and antioxidant biological investigations of *M*. *sinaica* extracts.

## 2. Results and Discussion

### 2.1. GC/MS Analysis of the Essential Oil and Lipophilic Extract

**Figure 1:**
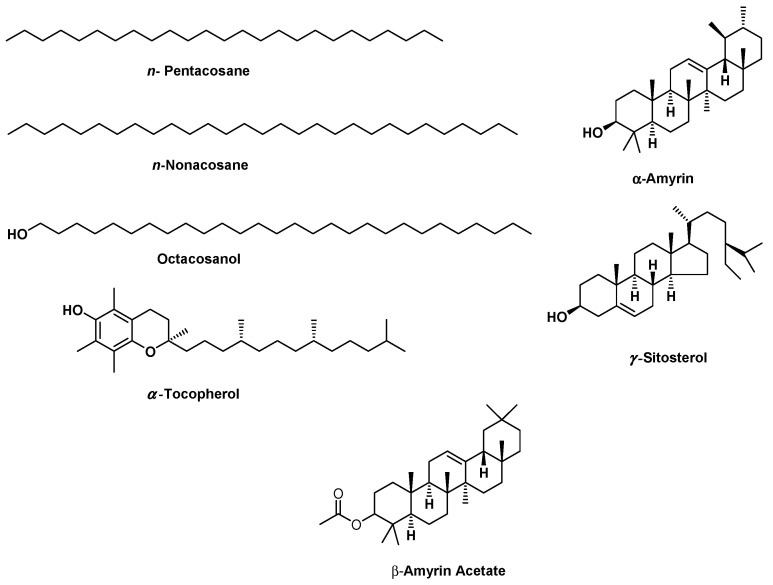
The chemical structures of the major components found in the essential oil and lipophilic extract of *Moricandia sinaica* aerial parts using GC/MS analysis.

**Figure 2:**
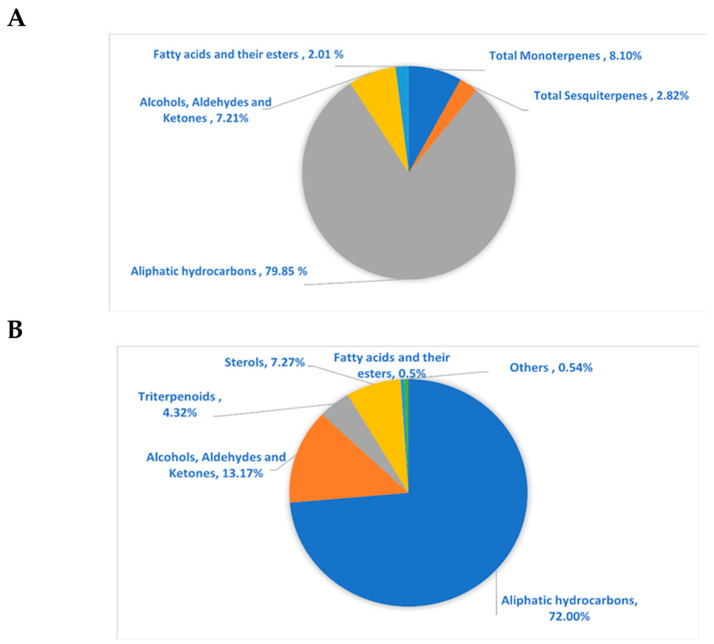
The pie charts show the percentage distribution of volatile components within (**A**) essential oil and (**B**) lipophilic extract of *M. sinaica* aerial parts.

The GC/MS analysis investigation of the lipophilic extract and essential oil of *M. sinaica* is shown in Table 1 (Supplementary Figures S1 and S2). The evaluation of the lipophilic extract and essential oil contents revealed the identification of 15 and 22 chemicals, representing 97.80% and 99.90%, respectively. Both the lipophilic extract and the essential oil were found to be rich in aliphatic hydrocarbons, accounting for 72.00% and 79.85%, respectively, where *n*-pentacosane (78.01%) is the major hydrocarbon in the essential oil and *n*-nonacosane (65.66%) is the major hydrocarbon in the lipophilic extract. In the lipophilic extract, octacosanol (12.67%), *γ*-sitosterol (6.60%), *α*-amyrin (3.51%), *β*-amyrin acetate (0.81%), and *α*-tocopherol (0.54%) are the major constituents. In contrast, monoterpenes in the essential oil (8.10%) are represented as tricyclene, camphene, *ꞵ*-citronellene, octanal, linalool, and *α*-terpineol, in addition to sesquiterpenes (2.82%) such as *α*-cadinene, caryophylla-4(12),8(13)-dien-5*α*-ol, and *ꞵ*-eudesmol. The chemical structures of the major compounds as well as the percentage distribution of volatile components of the lipophilic extract and essential oil of *M. sinaica* are displayed in Figure 1 and Figure 2.

Prior studies on the chemical characteristics of *M. arvensis* aerial parts essential oil obtained from two areas in Algeria revealed the richness of the oil of the Southern Setif population with palmitic acid (13.2–12.9%) and phytol (7.9–10.5%). In comparison, the oil of the Northern population is rich with 3-butenylisothiocyanate and octadecanoic acid, 2-hydroxy-1,3-p [27]. Another report by Marrelli et al. revealed the n-hexane extract’s chemical composition of *M. arvensis* using GC/MS analysis. The findings revealed the existence of different fatty acids such as palmitic, stearic, and myristic acids, together with phytosterols such as *ꞵ*-Sitosterol, 22,24-dimethylcholesterol, and stigmasta-3,5-dien-7-one [28]. Comparing the current findings to the previously published findings, both the essential oil and the lipophilic extract revealed variations in the components and their relative quantities that might be utilized as a chemical fingerprint to assess the validity and to discriminate between the given oils or extracts.

### 2.2. Cytotoxic Activity Using SRB Assay

**Figure 3:**
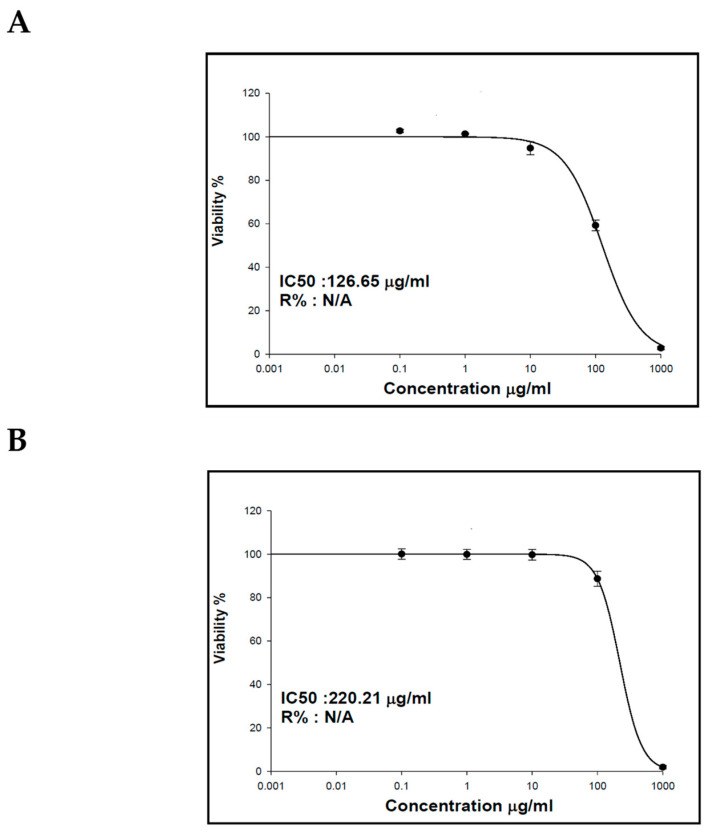
The effect of the (**A**) essential oil and (**B**) lipophilic extract of the aerial parts of *M. sinaica* on human liver cancer cells’ (HepG2) viability.

Previous studies on essential oils and lipophilic extracts of *M. sinaica* revealed their richness with potent secondary metabolites that have an effect on numerous cancer cells [23,29]. The cytotoxicity results of the essential oil and the lipophilic extract of *M. sinaica* aerial parts revealed their inhibitory activities toward human liver cancer cells (HepG2) with IC_50_ values of 126.65 and 220.21 µg/mL, respectively (Figure 3). In agreement with our results, GC/MS evaluation demonstrated the abundance of *n*-pentacosane in the essential oil of *M. sinaica* aerial parts (78.01%); it is a 25-carbon unbranched chain that was discovered in several essential oils with antimicrobial properties [30]. Additionally, it was reported to contain anticancer, antifungal, anti-inflammatory, antioxidant, and antiviral agents [31,32]. *α*-amyrin (3.51%) and *β*-amyrin acetate (0.81%) are also found to have antioxidant, antimicrobial, anti-inflammatory, and anticancer properties [23]. Octacosanol and 1-Octacosanol were also found to account for 12.67% and 0.50%, respectively. Studies have shown that octacosanol is an antiangiogenic substance that inhibits angiogenesis. Octacosanol prevents neovascularization and the proliferation of endothelial cells [33].

### 2.3. Antioxidant Activity

Natural polyphenols from the Brassicaceae family are abundant, provide a wide range of health implications, and are known for their antioxidant effects [34]. That gives us an interest in exploring the essential oils’ and the lipophilic extract’s potential as antioxidants using DPPH and FRAP assays. The lipophilic extract in the DPPH assay showed an IC_50_ value of 2679 ± 128.13 µg/mL as compared to the Trolox reference drug (IC_50_ = 4.94 ± 0.263 µg/mL). On the other hand, the lipophilic extract showed considerable antioxidant activity in the FRAP assay, expressed as 44.30 ± 3.73 µM Trolox equivalent/mg sample. The essential oil of *M. sinaica* aerial parts did not show any significant results in both assays. Previous reports regarding the biological activity of different *Moricandia* species revealed that *M. nitens* green synthesized GNPs showed substantial anti-*Helicobacter pylori* and anticancer activity against HepG2 and HCT-116 and were reported to be an effective α-glucosidase inhibitor [35]. Moreover, the methanolic extract of *M. arvensis* exhibited potent lipase-inhibitory activity and antioxidant activity [28].

### 2.4. Molecular Docking

**Figure 4:**
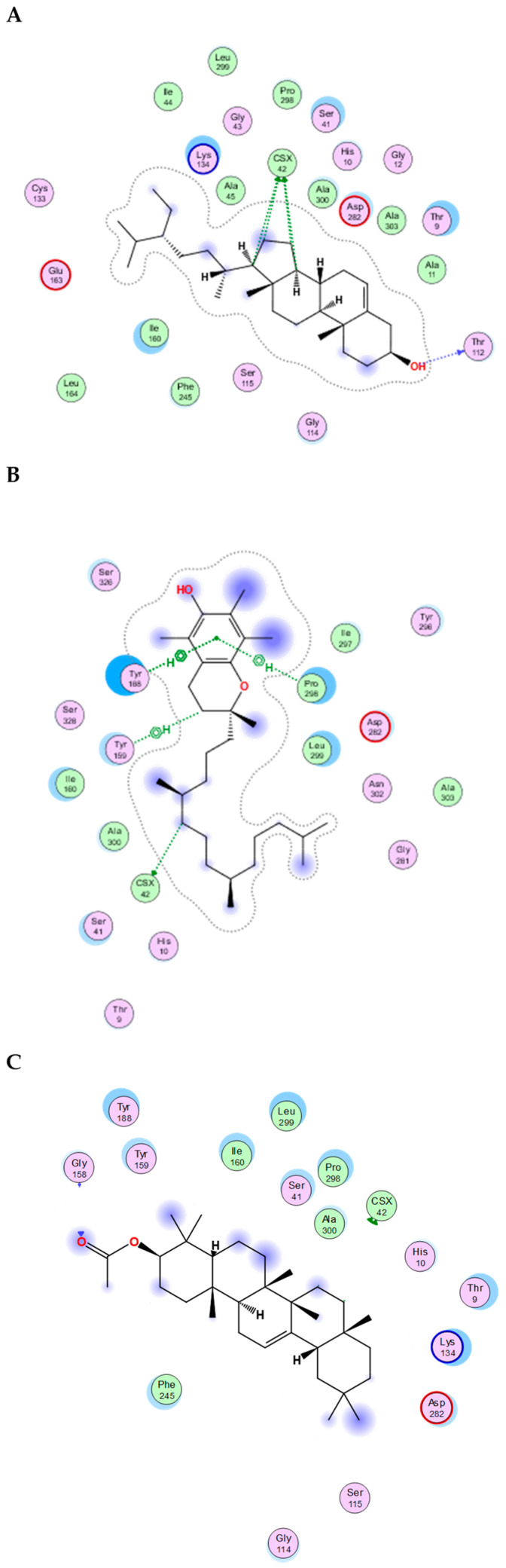
2D binding modes of *γ*-Sitosterol (**A**), *α*-Tocopherol (**B**), *ꞵ*-Amyrin Acetate (**C**) to the active binding sites of NADPH oxidase.

**Figure 5:**
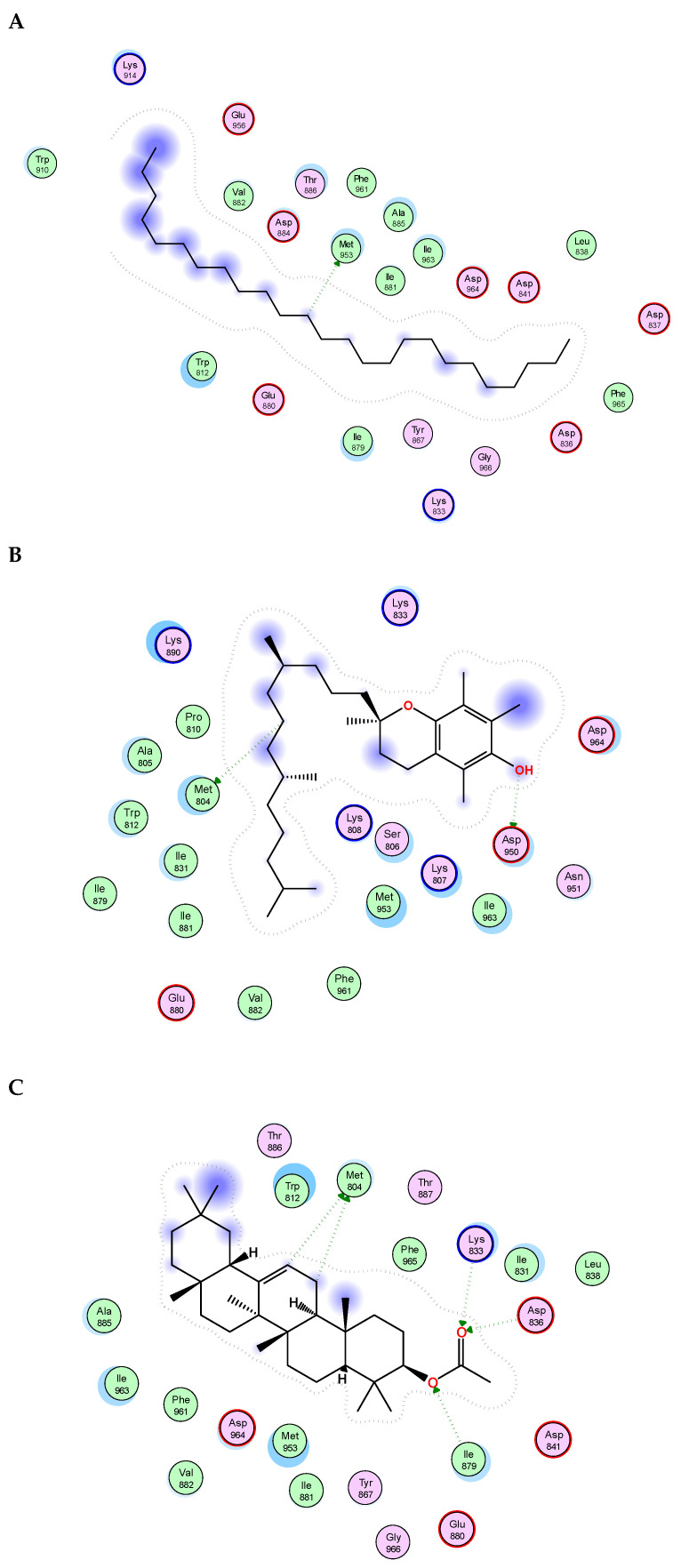
2D binding modes of n- pentacosane (**A**), *α*-Tocopherol (**B**), *ꞵ*-Amyrin Acetate (**C**) to the active binding sites of Phosphoinositide-3 kinase (PI3K).

**Figure 6:**
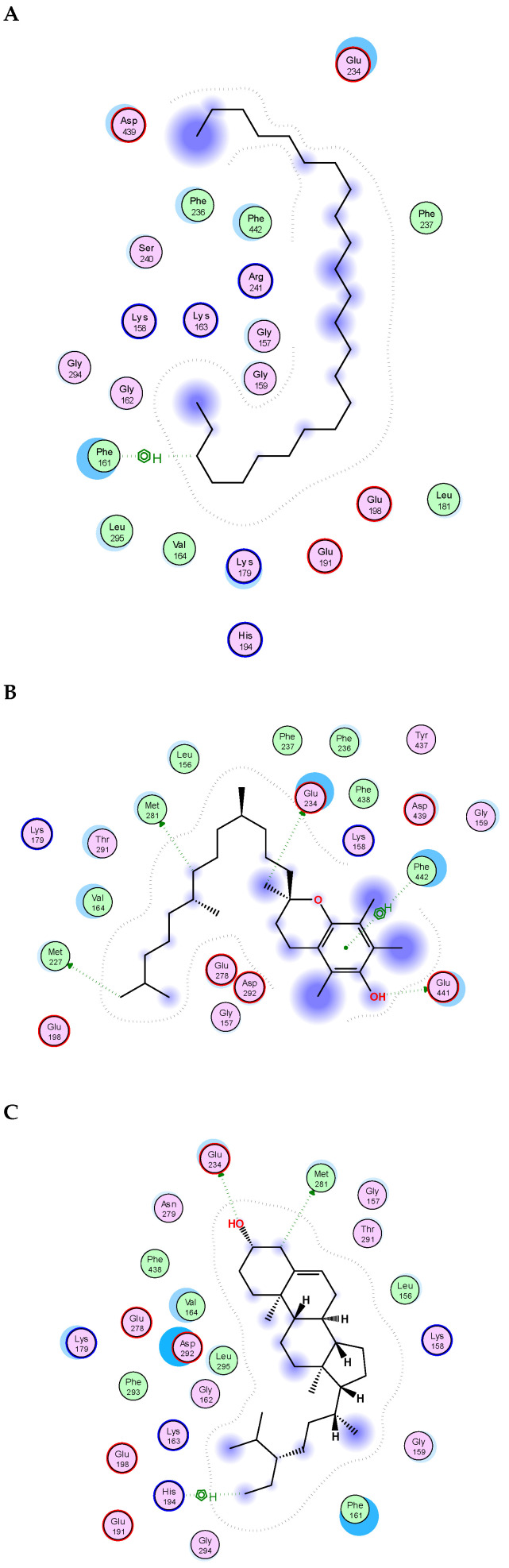
2D binding modes of n- pentacosane (**A**), *α*-Tocopherol (**B**), γ-sitosterol (**C**) to the active binding sites of Protein kinase B (AKT).

The essential oil and lipophilic extract of *M. sinaica*, which has potential cytotoxic and antioxidant properties, led us to perform a docking investigation of the major components against the enzymes NADPH oxidase, phosphoinositide-3 kinase (PI3K), and Akt, also known as protein kinase B (PKB). This research aimed to determine the probable binding mechanisms by which the major investigated metabolites may function. Therefore, the major components were docked into the NADPH oxidase, phosphoinositide-3 kinase (PI3K), and Akt 3D coordinates that were downloaded from the protein data bank using the following PDB IDs: 2cdu, 1E90, and 3cwq, respectively. Re-docking each co-crystallized ligand into its associated active site enabled us to confirm the docking parameters applied. The estimated RMSD values between the co-crystallized pose and the docked pose were 1.02, 0.97, and 1.03 Å NADPH oxidase, phosphoinositide-3 kinase (PI3K), and Akt, respectively, helping to ensure the docking technique is valid. The co-crystallized ligand’s re-docking resulted in docking scores of −11.3, −12.1, and −13.4 Kcal/mole for NADPH oxidase, phosphoinositide-3 kinase (PI3K), and Akt, respectively. The docking of the main metabolites to the three enzymes showed acceptable results corresponding to those of the reference compounds. Interestingly, in the docking with NADPH oxidase, ꞵ-amyrin acetate, α-tocopherol and γ-sitosterol were the best compounds, achieving docking scores of −14.19, −13.12, and −12.65 Kcal/Mol, respectively (Figure 4). Similarly, in the docking with PI3K, n-pentacosane, α-tocopherol and ꞵ-amyrin acetate were the best compounds, achieving docking scores of −13. 91, −14.15, and −14.05 Kcal/Mol, respectively (Figure 5). Worth noting, in the docking with AKT, n-pentacosane, α-tocopherol, and γ-sitosterol were the best compounds, achieving docking scores of −11. 9, −13.19, and −12.05 Kcal/Mol, respectively (Figure 6). The major compounds’ docking scores against the three proposed target enzymes are represented in Table 2. The major compounds’ interactions with the three enzymes are represented in Table 3, Table 4 and Table 5. The results of docking correlated with the results of the cytotoxic and antioxidant activities of the lipophilic extract of *M. sinaica*.

## 3. Materials and Methods

### 3.1. Plant Material

Leaves of *Moricandia sinaica* Boiss. (Bra) were obtained in February 2022 from South Sinai, Egypt 27°57′43.2″ N and 34°16′16.7″ E. The plant was thankfully recognized and verified by Dr. Mohammed El-Gebaly (Department of Botany, National Research Centre), Giza, Egypt. Voucher specimens were provided in the Pharmacognosy Department, Faculty of Pharmacy, Badr University in Cairo (Voucher specimen number: BUC-PHG-MS-10).

### 3.2. The Essential Oil Isolation

The leaves were finely chopped and hydro-distilled for 5 h with a Clevenger apparatus. The produced oil is yellowish orange in color with a fragrant odor; the yield was 0.21% (21 mg/100 g). It was collected and maintained at −4 °C in a tight, dark glass vial for GC/MS analysis.

### 3.3. Preparation of the Lipophilic Extract

Dry leaves (100 g) of *Moricandia sinaica* Boiss. were extracted three times with n-hexane. To obtain the dried residue of the lipophilic extract, the filtrate was fully evaporated in vacuo at 40 °C until dryness, and (2.41 g) of the lipophilic extract was obtained. The lipophilic extract was kept in a tightly sealed container for subsequent examination.

### 3.4. Gas Chromatography–Mass Spectrometry (GC/MS)

A Shimadzu GCMS-QP 2010 chromatograph (Kyoto, Japan) with a DB-5 capillary column (30 m × 0.25 mm i.d. × 0.25 μm film thickness; Restek, Bellefonte, PA, USA) was used for gas chromatography/mass spectrometry (GC/MS) analysis. The oven temperature was set at 50 °C for 3 min (isothermal), programmed to 30 °C at a rate of 5 °C/min, and kept constant at 300 °C for 10 min (isothermal); the temperature of the injector was 280 °C. Helium was employed as the carrier gas, with a flow rate of 1.40 mL/min. Diluted samples (1% *v/v*) were injected at a split ratio of 15:1 in a volume of 1 μL. The following were the MS running specifications: 280 °C for the interface, 220 °C for the ion source, 70 eV for the EI mode, and 35–500 amu for the scan range.

### 3.5. Characterization of the Essential Oil and Lipophilic Extract Components

The volatile constituents were characterized based on their retention indices and fragmentation patterns matching with NIST Mass Spectral Library, Wiley library database and published in the literature [23,26,29,36,37,38,39]. Retention indices (RI) were estimated in comparison to homologous series of *n*-alkanes (C8-C30) injected under the same conditions.

### 3.6. Assessment of Cytotoxic Activity Using SRB Assay

Nawah Scientific Inc., (Mokatam, Cairo, Egypt) provided the Hepatocellular carcinoma (HepG2). Cells were cultured in DMEM media treated with 100 mg/mL of streptomycin, 100 units/mL of penicillin, and 10% heat-inactivated fetal bovine serum in a humidified, 5% (*v/v*) CO_2_ atmosphere at 37 °C. The SRB assay was employed to assess cell viability. In 96-well plates, aliquots of 100 μL cell suspension (5 × 10^3^ cells) were incubated in complete media for 24 h. Another aliquot of 100 μL of media containing different doses of drugs was used to treat the cells. Cells were fixed by replacing media with 150 μL of 10% TCA and incubated at 4 °C for 1 h, after 72 h of drug exposure. After removing the TCA solution, the cells were washed 5 times with distilled water. Aliquots of 70 μL SRB solution (0.4% *w/v*) were added and incubated in a dark place at room temperature for 10 min. Plates were washed with 1% acetic acid for 3 times before being let to air dry overnight. Then, 150 μL of TRIS (10 mM) was added to dissolve protein-bound SRB stain; with the use of a BMGLABTECH®- FLUO star Omega microplate reader (Ortenberg, Germany), the absorbance was determined at 540 nm [40]. 

### 3.7. Assessment of Antioxidant Activity

#### 3.7.1. DPPH Assay

Samples were prepared using DMSO at a concentration of 50 mg/mL. Then, from this stock, the oil was prepared at a concentration of 2 mg/mL in methanol, and the lipophilic extract was prepared at a concentration of 10 mg/mL in methanol. A stock solution of 20 μg/mL of Trolox was prepared in methanol, from which 5 concentrations were prepared, including 12.5, 7.5, 6.25, 2.5, and 1.25 μg/mL. DPPH (2,2-diphenyl-1-picryl-hydrazyl-hydrate) free radical assay was carried out according to the method of [41]. Briefly, at room temperature, 100 μL of freshly prepared DPPH reagent (0.1% in methanol) was added to 100 μL of the sample in 96-well plates (*n* = 6), which was incubated for 30 min in the dark. The resulting reduction in DPPH color intensity was measured at 540 nm after the incubation time. The data are displayed as means ± SD according to the following equation:

$$\mathrm{Percentage}\;\mathrm{inhibition}=\frac{\mathrm{Average}\;\mathrm{absorbance}\;\mathrm{of}\;\mathrm{blank}-\mathrm{average}\;\mathrm{absorbance}\;\mathrm{of}\;\mathrm{the}\;\mathrm{test}}{\mathrm{Average}\;\mathrm{absorbance}\;\mathrm{of}\;\mathrm{blank}}\times 100$$

The microplate reader FluoStar Omega was used for recording the results. The data were processed using Microsoft Excel^®^ and the IC_50_ value calculation was performed using Graph pad Prism 6® by converting the concentrations to their logarithmic value and selecting a non-linear inhibitor regression equation (log (inhibitor) vs. normalized response—variable slope equation).

#### 3.7.2. FRAP Assay

**Figure 7:**
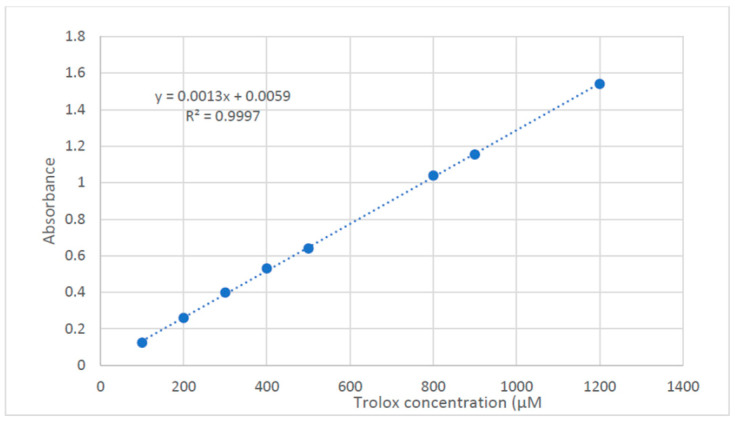
Dose response line for solution of Trolox.

Trolox Standard for FRAP assay Trolox stock solution of 3 mM in methanol was prepared, and the following dilutions were prepared at the concentrations of 1200, 1000, 800, 500, 400, 300, 200, and 100 μM. Samples were prepared at a concentration of 50 mg/mL in DMSO. Then, the oil was prepared at a concentration of 2 mg/mL in methanol, and the lipophilic extract was prepared at a concentration of 10 mg/mL in methanol. The assay was carried out in accordance with the method of Benzie et al. [42] with some modifications to be carried out in microplates. A freshly prepared TPTZ reagent (300 mM Acetate Buffer (PH = 3.6), 10 mM TPTZ in 40 mM HCl, and 20 mM FeCl_3_, in a ratio of 10:1:1 *v/v/v*, respectively). About 190 uL of the freshly prepared TPTZ reagent were mixed with 10 μL of the sample in 96-well plates (*n* = 3), the reaction was incubated at room temperature for 30 min in the dark. The resulting blue color was measured at 593 nm at the end of the incubation period. The data are represented as means ± SD. The microplate reader FluoStar Omega was used to record the results. The ferric-reducing ability of the samples is presented as μM TE/ mg sample using the linear regression equation obtained from the following calibration curve (linear dose-response curve of Trolox) (Figure 7).

### 3.8. Molecular Docking

Applying the software Molecular Operating Environment (MOE 2019.02), docking investigations were carried out [43,44]. The X-ray crystal structures of NADPH oxidase, phosphoinositide-3 kinase (PI3K), and Akt, known as protein kinase B (PKB) were obtained from the protein data bank www.pdb.org (accessed on 10 December 2022) using the following PDB IDs: 2CDU, 1E90, and 3CWQ, respectively [45,46,47]. Hydrogens and charges of the receptors were adjusted through AMBER10: EHT implanted in MOE software. The relevant co-crystallized ligand is bound at the established binding sites of three enzymes. Five major compounds identified in the essential oil (*n*-pentacosane, camphene, *ꞵ*-citronellene, linalool, and *α*-terpineol) and six compounds from the lipophilic extract (*n*-nonacosane (65.66%), octacosanol, *γ*-sitosterol, *α*-amyrin and *β*-amyrin acetate, and *α*-tocopherol) were produced using the 2D builder of MOE2019 and transformed into 3D structures with the same software. Utilizing the triangular matcher and London dg, the compounds were docked onto the three enzyme binding sites as placement and scoring methods, respectively. Finally, MOE produced 2D interaction diagrams to analyze the docking observations.

## 4. Conclusions

In the present research, the chemical investigation of *Moricandia sinaica* lipophilic extract and essential oil was analyzed using the GC/MS technique. The results revealed that both are rich in aliphatic hydrocarbons, where *n*-pentacosane (78.01%), and *n*-nonacosane (65.66%) are the major hydrocarbons in the essential oil and the lipophilic extract. It is worth noting that *γ*-sitosterol, *α*-amyrin, *β*-amyrin acetate, and *α*-tocopherol are the main important constituents in the lipophilic extract that correlate with its antioxidant activity using DPPH and FRAP assays. In addition, monoterpenes and sesquiterpenes are present in the essential oil, which correlates with its cytotoxic activity against HEPG-2 cells. The biological investigations were supported by the molecular docking study and interactions of the major constituents with NADPH oxidase, phosphoinositide-3 kinase (PI3K), and Akt, known as protein kinase B (PKB) enzymes. Accordingly, the lipophilic extract and essential oil of *M. sinaica* would be a promising alternative for the production of cytotoxic and antioxidant agents, supported by further in vivo investigations as well as pharmacodynamic and pharmacokinetic studies.

## Figures and Tables

**Table 1:** Chemical composition (%) of the essential oil and the lipophilic extract identified in *Moricandia sinaica* aerial parts using GC/MS analysis.

No.	Rt_(min)_	Compound	RI_Exp_. ^a^	RI_Lit_ ^b^	Molecular Formula	Content (%)	
						MSH	MSO
1	3.44	1-Methyl-1,3-cyclohexadiene	765	770	C_7_H_10_	-	1.03
2	4.33	2-Hexanol	800	801	C_6_H_14_O	-	0.95
3	4.98	Ethylcyclohexane	826	827	C_8_H_16_	-	0.48
4	5.41	2-Hexen-1-al	843	847	C_6_H_12_O	-	0.32
5	5.47	2-Hexenal, (*E*)-	845	846	C_6_H_10_O	-	0.62
6	5.62	3(*Z*)-Hexen-1-ol	851	851	C_6_H_12_O	-	2.09
7	6.20	2-Heptanone	874	880	C_7_H_14_O	-	0.66
8	6.39	Santene	881	884	C_9_H_14_	-	0.33
9	6.83	Heptanal	899	900	C_7_H_14_O	-	0.91
10	7.30	Tricyclene	915	914	C_10_H_16_	-	1.79
11	8.12	Camphene	942	941	C_10_H_16_	-	1.15
12	8.18	*ꞵ*-Citronellene	943	947	C_10_H_20_O	-	0.82
13	9.88	Octanal	1000	1002	C_8_H_16_O	-	1.66
14	13.00	Linalool	1099	1099	C_10_H_18_O	-	1.97
15	16.06	*α*-Terpineol	1199	1191	C_10_H_18_O	-	1.28
16	20.34	*δ*-Terpinyl acetate	1348	1340	C_12_H_20_O_2_	-	1.09
17	24.96	*α*-Cadinene	1530	1538	C_15_H_24_	-	0.49
18	27.54	Caryophylla-4(12),8(13)-dien-5*α*-ol	1642	1640	C_15_H_24_O	-	1.17
19	28.03	*ꞵ*-Eudesmol	1659	1654	C_15_H_24_O	-	1.16
20	31.84	Decanoic acid butyl ester	1820	1821	C_14_H_28_O_2_	-	1.21
21	43.38	Docosahexaenoic acid methyl ester	2478	2470	C_23_H_34_O_2_	0.5	-
22	43.66	*n*-Pentacosane	2496	2500	C_25_H_52_	0.4	78.01
23	46.76	*n*-Heptacosane	2696	2700	C_27_H_56_	2.33	-
24	47.71	Lignoceric acid methyl ester	2712	2732	C_25_H_50_O_2_	-	0.80
25	48.22	*n*-Octacosane	2796	2800	C_28_H_58_	0.89	-
26	49.69	*n*-Nonacosane	2900	2900	C_29_H_60_	65.66	-
27	51.00	*n*-Triacontane	2996	3000	C_30_H_62_	0.66	-
28	51.83	2-Methyltriacontane	3058	3060	C_31_H_64_	0.64	-
29	52.18	1-Octacosanol	3084	3074	C_28_H_58_O	0.50	-
30	52.33	*n*-Hentriacontane	3096	3100	C_31_H_64_	1.42	-
31	52.46	Octacosanol	3106	3110	C_28_H_58_O	12.67	-
32	53.03	*α*-Tocopherol	3150	3149	C_29_H_50_O_2_	0.54	-
33	54.51	5*α*-Stigmast-22-en-3*β*-ol	3257	3253	C_29_H_50_O	0.67	-
34	55.95	γ-Sitosterol	3350	3351	C_29_H_50_O	6.60	-
35	56.24	α-Amyrin	3367	3371	C_30_H_50_O	3.51	-
36	56.94	*β*-Amyrin acetate	3430	3437	C_32_H_52_O_2_	0.81	-
Total Monoterpenes						-	8.1
Total Sesquiterpenes						-	2.82
Aliphatic hydrocarbons						72.00	79.85
Alcohols, Aldehydes and Ketones						13.17	7.21
Triterpenoids						4.32	-
Sterols						7.27	-
Fatty acids and their esters						0.50	2.01
Others						0.54	-
Total identified %						97.80	99.90

Compounds are listed in the order of elution on the DB-5 GC column. The compound’s mass spectral data (MS) and retention indices (RI) were compared to those in the NIST Mass Spectral Library 2011), Wiley Registry of Mass Spectral Data 8th edition, and the literature for identification [22,23,24,25,26]. ^a^ Retention index calculated experimentally on DB-5 GC column in comparison to n-alkane series (C8–C28). ^b^ Published retention indices.

**Table 2:** Docking scores of major identified constituents in the essential oil and lipophilic extract of *M. sinaica* against three target enzymes: NADPH oxidase, Phosphoinositide-3 kinase, and Protein kinase B.

Compound Name	Docking Scores kcal/mole		
	NADPH Oxidase 2CDU	Phosphoinositide-3 Kinase (PI3K) 1E90	Protein Kinase B, AKT 3CWQ
*n*-Pentacosane	−10.83	−13.91	−11.9
Camphene	−6.61	−7.22	−6.96
*ꞵ*-Citronellene	−7.03	−7.92	−7.62
Linalool	−7.39	−8.25	−7.80
*α*-Terpineol	−9.20	−8.19	−8.32
n-Nonacosane	−8.96	−8.83	−7.56
*α*-Amyrin	−12.01	−13.37	−11.19
Octacosanol	−10.76	−10.02	−11.41
*γ*-Sitosterol	−12.65	−12.44	−12.05
*α*-Tocopherol	−13.12	−14.15	−13.19
*ꞵ*-Amyrin Acetate	−14.19	−14.05	−11.70

**Table 3:** Docking results of major identified constituents in the essential oil and lipophilic extract of *M. sinaica* on NADPH.

Compound Name	Ligand	Receptor	Interaction	Distance	E (kcal/mol)
*n*-Pentacosane	C-17	6-ring Tyr-159	(A) H-pi	4.73	−0.2
	C-23	6-ring Tyr-159	(A) H-pi	4.21	−0.5
	C-41	6-ring Phe-245	(A) H-pi	4.93	−0.2
Camphene	C-5	SG Csx-42	(A) H-donor	4.16	−0.2
*ꞵ*-Citronellene	C-21	SG Cys-133	(A) H-donor	4.19	−0.2
	C-17	6-ring Phe-245	(A) H-pi	3.57	−0.3
Linalool	C-1	SG Csx-42	(A) H-donor	3.96	−0.2
	O-19	OD2 Asp-282	(A) H-donor	3.07	−2.0
	C-9	6-ring Phe-245	(A) H-pi	4.80	−0.3
*α*-Terpineol	C-9	SG Csx-42	(A) H-donor	4.14	−0.3
	O-28	OD1 Asp-282	(A) H-donor	2.93	−1.5
*n*-Nonacosane	C-1	SD Met-33	(A) H-donor	4.20	−0.2
	C-5	SD Met-33	(A) H-donor	4.08	−0.2
	C-8	SD Met-33	(A) H-donor	4.13	−0.2
	C-11	SD Met-33	(A) H-donor	4.30	−0.2
	C-41	SG CSX-42	(A) H-donor	4.49	−0.2
	C-68	6-ring Tyr-159	(A) H-pi	4.17	−0.5
	C-74	6-ring Tyr-159	(A) H-pi	4.68	−0.2
*α*-Amyrin	C-46	SG Csx-42	(A) H-donor	4.13	−0.2
	O-50	O Thr-112	(A) H-donor	2.90	−0.4
Octacosanol	C-1	SD Met-33	(A) H-donor	3.90	−0.3
	C-40	SG Csx-42	(A) H-donor	3.93	−0.2
	C-43	SG Csx-42	(A) H-donor	3.90	−0.2
	O-86	N Val-81	(A) H-acceptor	3.11	−2.3
	C-64	6-ring Tyr-159	(A) H-pi	4.04	−0.3
	C-70	6-ring Tyr-159	(A) H-pi	4.38	−0.2
*γ*-Sitosterol	C-21	SG Csx-42	(A) H-donor	4.46	−0.2
	C-29	SG Csx-42	(A) H-donor	4.25	−0.2
	C-31	SG Csx-42	(A) H-donor	3.83	−0.2
	C-34	SG Csx-42	(A) H-donor	4.14	−0.2
	O-74	O Thr-112	(A) H-donor	2.97	−0.2
*α*-Tocopherol	C-44	SG Csx-42	(A) H-donor	3.99	−0.2
	C-9	6-ring Tyr-159	(A) H-pi	4.21	−0.2
	6-ring	CE2 Tyr-188	(A) pi-H	3.63	−0.2
	6-ring	OH Tyr-188	(A) pi-H	3.59	−0.3
	6-ring	CD Pro-298	(A) pi-H	4.51	−0.4
*ꞵ*-Amyrin Acetate	C-24	SG Csx-42	(A) H -donor	4.19	−0.2
	C-61	SG Csx-42	(A) H -donor	4.17	−0.2
	O-81	CA Gly-158	(A) H-acceptor	3.30	−0.4

**Table 4:** Docking results of major identified constituents in the essential oil and lipophilic extract of *M. sinaica* on Phosphoinositide-3 kinase (PI3K).

Compound Name	Ligand	Receptor	Interaction	Distance	E (kcal/mol)
*n*-Pentacosane	C 35	SD Met-953	(A) H-donor	3.59	−0.2
Camphene	No binding				
*ꞵ*-Citronellene	C-7	SD Met-953	(A) H-donor	3.73	−0.2
	C-12	SD Met-953	(A) H-donor	3.81	0.2
	C-17	SD Met-953	(A) H-donor	4.03	−0.2
Linalool	C-12	SD Met-953	(A) H-donor	3.93	−0.3
	O-19	O Glu-880	(A) H-donor	2.84	−1.6
	O-19	CD1 Ile-831	(A) H-acceptor	3.55	−0.2
	O-19	CG2 Ile-879	(A) H-acceptor	3.49	−0.2
	O-19	CD1 Ile-881	(A) H-acceptor	3.71	−0.2
*α*-Terpineol	O-28	OD1 Asp-964	(A) H-donor	3.18	−0.5
	O-28	OD2 Asp-964	(A) H-donor	3.00	−0.3
	O-28	NZ Lys-833	(A) H-acceptor	2.72	−7.0
	C-12	6-ring Tyr-867	(A) H-pi	4.59	−0.2
*n*-Nonacosane	C-41	SD Met-953	(A) H-donor	3.87	−0.2
	C-44	SD Met-953	(A) H-donor	3.80	−0.2
	C-47	SD Met-953	(A) H-donor	3.95	−0.2
*α*-Amyrin	C-15	SD Met-804	(A) H-donor	4.33	−0.2
	C-50	O Val-882	(A) H-donor	2.56	−2.2
	C-50	CB Ala-885	(A) H-acceptor	3.20	−0.4
Octacosanol	O-86	OG Ser-806	(A) H-donor	2.86	−0.8
	O-86	NZ Lys-807	(A) H-acceptor	3.10	−7.5
*γ*-Sitosterol	C-16	SD Met-804	(A) H-donor	4.19	−0.2
	C-21	SD Met-804	(A) H-donor	4.17	−0.2
	C-64	SD Met-953	(A) H-donor	4.20	−0.2
*α*-Tocopherol	O-19	OD2 Asp-950	(A) H-donor	2.76	−3.7
	C-47	SD Met-804	(A) H-donor	3.99	−0.2
*ꞵ*-Amyrin Acetate	C-82	SD Met-804	(A) H-donor	3.90	−0.2
	C-85	SD Met-804	(A) H-donor	3.76	−0.2
	C-41	CD1 Ile-879	(A) H-acceptor	3.84	−0.2
	C-81	CD Lys-833	(A) H-acceptor	3.26	−0.8
	C-81	NZ Lys-833	(A) H-acceptor	3.61	−2.5
	C-81	CB Asp-836	(A) H-acceptor	3.10	−0.2

**Table 5:** Docking results of major identified constituents in the essential oil and lipophilic extract of *M. sinaica* on Protein kinase B.

Compound Name	Ligand	Receptor	Interaction	Distance	E (kcal/mol)
Co Crys. Ligand	N22 26	OG1 THR291	(A) H-donor	3.16	−1.2
	N8 15	N ALA 230	(A) H-acceptor	2.99	−5.4
*n*-Pentacosane	C 68	6-ring PHE 161	(A) H-pi	5.07	−0.2
Camphene	C 13	5-ring HIS 194	(A) H-pi	4.48	−0.2
	C 20	6-ring PHE 161	(A) H-pi	4.23	−0.3
*ꞵ*-Citronellene	No binding				
Linalool	O 19	OE1 GLU198	(A) H-donor	2.82	−1.6
	O 19	NZ LYS 179	(A) H-acceptor	3.08	−0.6
*α*-Terpineol	O 28	OG1 THR 195	(A) H-donor	2.94	−0.2
	C 9	6-ring PHE 161	(A) H-pi	4.96	−0.3
*n*-Nonacosane	No binding				
*α*-Amyrin	C 9	SD MET 281	(A) H-donor	4.09	−0.2
Octacosanol	O 86	O SER 240	(A) H-donor	2.91	−1.6
	C 79	6-ring PHE 161	(A) H-pi	5.00	−0.2
	C 79	5-ring HIS 194	(A) H-pi	4.62	−0.2
*γ*-Sitosterol	C 6	SD MET 281	(A) H-donor	4.02	−0.3
	O 74	OE1 GLU 234	(A) H-donor	3.17	−0.3
	C 60	5-ring HIS 194	(A) H-pi	4.37	−0.4
*α*-Tocopherol	O 19	OE1 GLU 441	(A) H-donor	3.08	−2.4
	C 32	OE2 GLU 234	(A) H-donor	3.86	−0.2
	C 50	SD MET 281	(A) H-donor	3.90	−0.2
	C 78	SD MET 227	(A) H-donor	3.90	−0.2
	6-ring	CZ PHE 442	(A) pi-H	3.83	−0.7
*ꞵ*-Amyrin Acetate	C 4	OD2 ASP 439	(A) H-donor	3.57	−0.4
	C 77	O TYR 437	(A) H-donor	3.32	−0.4

## Data Availability

Data are available upon request from the first author.

## Supplementary Materials

The following supporting information can be downloaded at: https://www.mdpi.com/article/10.3390/molecules28052193/s1, Figure S1. GC-chromatogram of *Moricandia sinaica* lipophilic extract; Figure S2. GC-chromatogram of *Moricandia sinaica* essential oil.

## References

[1] Kinghorn A.D., Pan L., Fletcher J.N., Chai H. (2011). The Relevance of Higher Plants in Lead Compound Discovery Programs. J. Nat. Prod..

[2] Aly S.H., Elissawy A.M., Fayez A.M., Eldahshan O.A., Elshanawany M.A., Singab A.N.B. (2020). Neuroprotective Effects of *Sophora Secundiflora*, *Sophora Tomentosa* Leaves and Formononetin on Scopolamine-Induced Dementia. Nat. Prod. Res..

[3] El-Nashar H.A.S., Aly S.H., Ahmadi A., El-Shazly M. (2021). The Impact of Polyphenolics in the Management of Breast Cancer: Mechanistic Aspects and Recent Patents. Recent Pat. Anticancer. Drug Discov..

[4] Ads E.N., Hassan S.I., Rajendrasozhan S., Hetta M.H., Aly S.H., Ali M.A. (2022). Isolation, Structure Elucidation and Antimicrobial Evaluation of Natural Pentacyclic Triterpenoids and Phytochemical Investigation of Different Fractions of *Ziziphus spina-christi* (L.) Stem Bark Using LCHRMS Analysis. Molecules.

[5] Aly S.H., Elissawy A.M., Allam A.E., Farag S.M., Eldahshan O.A., Elshanawany M.A., Singab A.N.B. (2021). New Quinolizidine Alkaloid and Insecticidal Activity of *Sophora secundiflora* and *Sophora tomentosa* against *Culex pipiens* (Diptera: Culicidae). Nat. Prod. Res..

[6] Farnsworth N.R., Akerele O., Bingel A.S. (1987). Medicinal Plants in Therapy. J. Ethnopharmacol..

[7] Cragg G.M., Newman D.J. (2013). Natural Products: A Continuing Source of Novel Drug Leads. Biochim. Biophys. Acta (BBA)-Gen. Subj..

[8] Atanasov A.G., Waltenberger B., Pferschy-Wenzig E.-M., Linder T., Wawrosch C., Uhrin P., Temml V., Wang L., Schwaiger S., Heiss E.H. (2015). Discovery and Resupply of Pharmacologically Active Plant-Derived Natural Products: A Review. Biotechnol. Adv..

[9] Saber F.R., Munekata P.E.S., Rizwan K., El-nashar H.A.S., Fahmy N.M., Aly S.H., El-shazly M., Bouyahya A., Lorenzo J.M. (2023). Family Myrtaceae: The Treasure Hidden in the Complex/Diverse Composition. Crit. Rev. Food Sci. Nutr..

[10] Aly S.H., Elissawy A.M., Salah D., Alfuhaid N.A., Zyaan O.H., Mohamed H.I., Singab A.N.B., Farag S.M. (2023). Phytochemical Investigation of Three Cystoseira Species and Their Larvicidal Activity Supported with In Silico Studies. Mar. Drugs.

[11] Abdel Razek M.M.M., Moussa A.Y., El-Shanawany M.A., Singab A.N.B. (2020). A New Phenolic Alkaloid from *Halocnemum Strobilaceum* Endophytes: Antimicrobial, Antioxidant and Biofilm Inhibitory Activities. Chem. Biodivers..

[12] Vaughn S.F., Berhow M.A. (2005). Glucosinolate Hydrolysis Products from Various Plant Sources: PH Effects, Isolation, and Purification. Ind. Crop. Prod..

[13] Warwick S.I., Francis A., Gugel R.K. (2009). Guide to Wild Germplasm of Brassica and Allied Crops (Tribe Brassiceae, Brassicaceae). Canada Agric. Agri-Food Canada.

[14] Perfectti F., Gómez J.M., González-Megías A., Abdelaziz M., Lorite J. (2017). Molecular Phylogeny and Evolutionary History of Moricandia DC (Brassicaceae). PeerJ.

[15] Kilian B., Mammen K., Millet E., Sharma R., Graner A., Salamini F., Hammer K., Ozkan H., Kole C. (2011). Aegilops. Wild Crop Relatives, Genomic and Breeding Resources Cereals.

[16] Skandrani I., Bouhlel I., Limem I., Boubaker J., Bhouri W., Neffati A., Sghaier M.B., Kilani S., Ghedira K., Ghedira-Chekir L. (2009). Moricandia Arvensis Extracts Protect against DNA Damage, Mutagenesis in Bacteria System and Scavenge the Superoxide Anion. Toxicol. Vitr..

[17] Le Floc’h E. (1983). Contribution à Une Étude Ethnobotanique de La Flore Tunisienne.

[18] Skandrani I., Ben Sghaier M., Neffati A., Boubaker J., Bouhlel I., Kilani S., Mahmoud A., Ghedira K., Chekir-Ghedira L. (2007). Antigenotoxic and Free Radical Scavenging Activities of Extracts from Moricandia Arvensis. Drug Chem. Toxicol..

[19] Skandrani I., Limem I., Neffati A., Boubaker J., Sghaier M.B., Bhouri W., Bouhlel I., Kilani S., Ghedira K., Chekir-Ghedira L. (2010). Assessment of Phenolic Content, Free-Radical-Scavenging Capacity Genotoxic and Anti-Genotoxic Effect of Aqueous Extract Prepared from *Moricandia Arvensis* Leaves. Food Chem. Toxicol..

[20] Arif I.A., Bakir M.A., Khan H.A., Al Farhan A.H., Al Homaidan A.A., Bahkali A.H., Al Sadoon M., Shobrak M. (2010). Application of RAPD for Molecular Characterization of Plant Species of Medicinal Value from an Arid Environment. Genet. Mol. Res..

[21] El-Mekkawy S., Shahat A.A., Alqahtani A.S., Alsaid M.S., Abdelfattah M.A.O., Ullah R., Emam M., Yasri A., Sobeh M. (2020). A Polyphenols-Rich Extract from *Moricandia Sinaica* Boiss. Exhibits Analgesic, Anti-Inflammatory and Antipyretic Activities in Vivo. Molecules.

[22] Radulović N.S., Dordević N.D. (2011). Steroids from Poison Hemlock (*Conium maculatum* L.): A GC-MS Analysis. J. Serbian Chem. Soc..

[23] Aly S.H., Elissawy A.M., Eldahshan O.A., Elshanawany M.A., Singab A.N.B. (2020). Phytochemical Investigation Using GC/MS Analysis and Evaluation of Antimicrobial and Cytotoxic Activities of the Lipoidal Matter of Leaves of *Sophora Secundiflora* and *Sophora Tomentosa*. Arch. Pharm. Sci. Ain Shams Univ..

[24] Jamalova D.N., Gad H.A., Akramov D.K., Tojibaev K.S., Al Musayeib N.M., Ashour M.L., Mamadalieva N.Z. (2021). Discrimination of the Essential Oils Obtained from Four Apiaceae Species Using Multivariate Analysis Based on the Chemical Compositions and Their Biological Activity. Plants.

[25] Al-Sayed E., Gad H.A., El-Kersh D.M. (2021). Characterization of Four Piper Essential Oils (GC/MS and ATR-IR) Coupled to Chemometrics and Their Anti- Helicobacter Pylori Activity. ACS Omega.

[26] Aly S.H., Eldahshan O.A., Al-rashood S.T., Binjubair F.A., El Hassab M.A., Eldehna W.M., Acqua S.D., Zengin G. (2022). Chemical Constituents, Antioxidant, and Enzyme Inhibitory Activities Supported by In-Silico Study of n-Hexane Extract and Essential Oil of Guava Leaves. Molecules.

[27] Zeraib A., Ramdani M., Lograda T., Chalard P., Figueredo G. (2011). Chemical Composition and Antimicrobial Activity of Essential Oil of *Moricandia Arvensis* L. (DC.). Asian J. Plant Sci..

[28] Marrelli M., Morrone F., Gambacorta L., Argentieri M.P., Conforti F., Avato P. (2018). Phytochemical and Biological Profile of *Moricandia arvensis* (L.) DC.: An Inhibitor of Pancreatic Lipase. Molecules.

[29] El-Nashar H.A.S., Eldehna W.M., Al-Rashood S.T., Alharbi A., Eskandrani R.O., Aly S.H. (2021). GC/MS Analysis of Essential Oil and Enzyme Inhibitory Activities of *Syzygium Cumini* ( Pamposia ) Grown in Docking Studies. Molecules.

[30] Turgumbayeva A., Ustenova G., Datkhayev U., Rahimov K., Abramavicius S., Tunaityte A., Zhakipbekov K., Kozhanova K., Tulemissov S., Ustenova O. (2020). Safflower (*Carthamus tinctorius* L.) a Potential Source of Drugs against Cryptococcal Infections, Malaria and Leishmaniasis. Phyton.

[31] Alamery S.F., Algaraawi N.L. (2020). Phytochemical Profile and Antifungal Activity of Stems and Leaves Methanol Extract from the *Juncus Maritimus* Linn. Juncaceae Family against Some Dermatophytes Fungi. AIP Conf. Proc..

[32] Gad H.A., Mukhammadiev E.A., Zengen G., Musayeib N.M.A., Hussain H., Ware I.B., Ashour M.L., Mamadalieva N.Z. (2022). Chemometric Analysis Based on GC-MS Chemical Profiles of Three Stachys Species from Uzbekistan and Their Biological Activity. Plants.

[33] Thippeswamy G., Sheela M.L., Salimath B.P. (2008). Octacosanol Isolated from Tinospora Cordifolia Downregulates VEGF Gene Expression by Inhibiting Nuclear Translocation of NF-<kappa>B and Its DNA Binding Activity. Eur. J. Pharmacol..

[34] Braham H., Mighri Z., Jannet H.B., Matthew S., Abreu P.M. (2005). Antioxidant Phenolic Glycosides from Moricandia Arvensis. J. Nat. Prod..

[35] Soliman N.A., Ismail E.H., Abd El-Moaty H.I., Sabry D.Y., Khalil M.M.H. (2018). Anti-Helicobacter Pylori, Anti-Diabetic and Cytotoxicity Activity of Biosynthesized Gold Nanoparticles Using *Moricandia Nitens* Water Extract. Egypt. J. Chem..

[36] Adams R.P. (2007). Identification of Essential Oil Components by Gas Chromatography/Mass Spectroscopy.

[37] Aly S.H., Elissawy A.M., Eldahshan O.A., Elshanawany M.A., Singab A.N.B. (2020). Variability of the Chemical Composition of the Essential Oils of Flowers and the Alkaloid Contents of Leaves of *Sophora Secundiflora* and *Sophora Tomentosa*. J. Essent. Oil-Bearing Plants.

[38] NIST The National Institute of Standards and Technology (NIST) Chemistry WebBook NIST Standard Reference Database Number 69. http://Webbook.Nist.Gov/Chemistry/.

[39] Aly S.H., El-hassab M.A., Elhady S.S., Gad H.A. (2022). Comparative Metabolic Study of *Tamarindus Indica* L.’s Various Organs Based on GC/MS Analysis, In Silico and In Vitro Anti-Inflammatory and Wound Healing Activities. Plants.

[40] Skehan P., Storeng R., Scudiero D., Monks A., McMahon J., Vistica D., Warren J.T., Bokesch H., Kenney S., Boyd M.R. (1990). New Colorimetric Cytotoxicity Assay for Anticancer-Drug Screening. J. Natl. Cancer Inst..

[41] Boly R., Lamkami T., Lompo M., Dubois J., Guissou I.P. (2016). DPPH Free Radical Scavenging Activity of Two Extracts From. Int. J. Toxicol. Pharmacol. Res..

[42] Benzie I.F.F., Strain J.J. (1996). The Ferric Reducing Ability of Plasma (FRAP) as a Measure of “Antioxidant Power”: The FRAP Assay. Anal. Biochem..

[43] El Hassab M.A., Fares M., Amin M.K.A., Al-rashood S.T., Alharbi A., Eskandrani R.O., Alkahtani H.M., Eldehna W.M. (2021). Toward the Identification of Potential α-Ketoamide Covalent Inhibitors for SARS-CoV-2 Main Protease: Fragment-Based Drug Design and MM-PBSA Calculations. Processes.

[44] Vilar S., Cozza G., Moro S. (2008). Medicinal Chemistry and the Molecular Operating Environment (MOE): Application of QSAR and Molecular Docking to Drug Discovery. Curr. Top. Med. Chem..

[45] Lountos G.T., Jiang R., Wellborn W.B., Thaler T.L., Bommarius A.S., Orville A.M. (2006). The Crystal Structure of NAD(P)H Oxidase from *Lactobacillus Sanfranciscensis*: Insights into the Conversion of O2 into Two Water Molecules by the Flavoenzyme. Biochemistry.

[46] Walker E.H., Pacold M.E., Perisic O., Stephens L., Hawkins P.T., Wymann M.P., Williams R.L. (2000). Structural Determinants of Phosphoinositide 3-Kinase Inhibition by Wortmannin, LY294002, Quercetin, Myricetin, and Staurosporine. Mol. Cell.

[47] Forouhar F., Neely H., Seetharaman J., Mao L., Xiao R., Janjua H., Maglaqui M., Foote E.L., Lee D., Everett J.K. (2008). Crystal Structure of Chromosome Partitioning Protein (ParA) in Complex with ADP from *Synechocystis* sp.. Northeast Struct. Genom. Consort..

