# Supplementary material for: GC/MS Profiling of the Essential Oil and Lipophilic Extract of Moricandia sinaica Boiss. and Evaluation of Their Cytotoxic and Antioxidant Activities
Source: Molecules. 2023 Feb 27;28(5):2193. doi: 10.3390/molecules28052193 (PMC10004251; doi:10.3390/molecules28052193)
Supplement: Supplementary file 1 [file molecules-28-02193-s001.zip › molecules-2246894-supplementary.pdf]

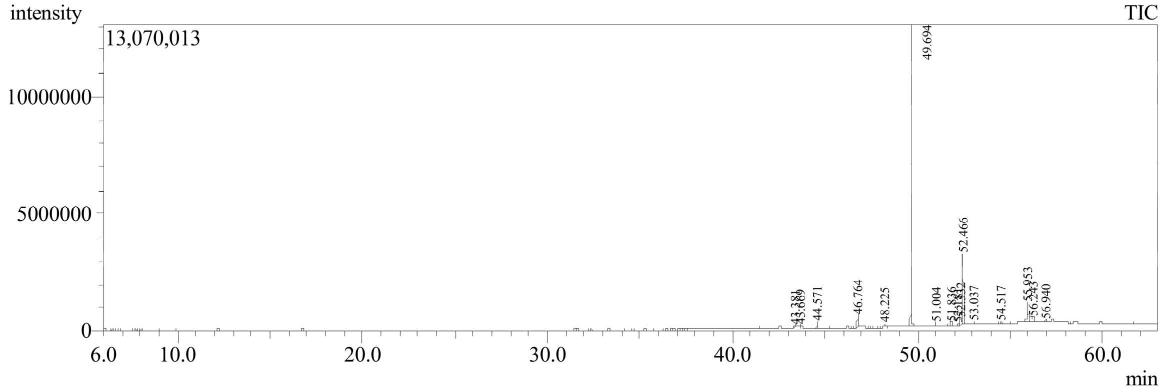

Figure S1. GC-chromatogram of *Moricandia sinaica* lipophilic extract.

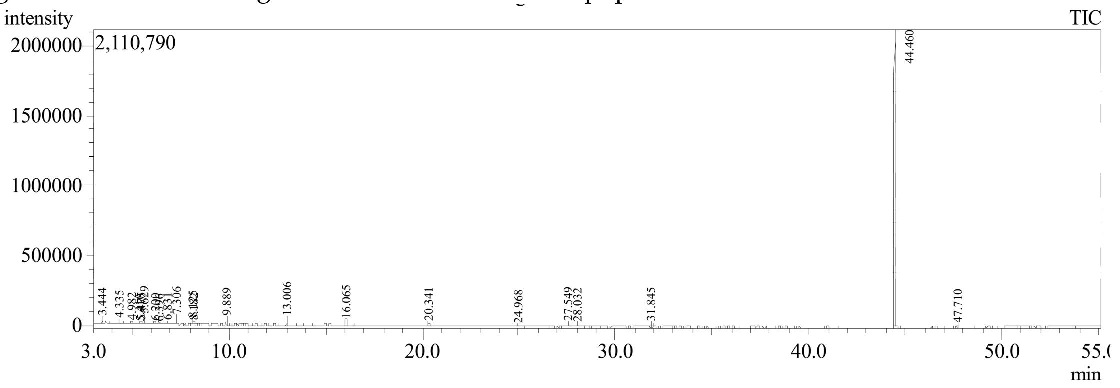

Figure S2. GC-chromatogram of *Moricandia sinaica* essential oil.
